# Current and prospective strategies for advancing the targeted delivery of CRISPR/Cas system via extracellular vesicles

**DOI:** 10.1186/s12951-023-01952-w

**Published:** 2023-06-08

**Authors:** Xiaowen Huang, Aifang Li, Peng Xu, Yangfan Yu, Shuxuan Li, Lina Hu, Shuying Feng

**Affiliations:** 1 Medical College, Henan University of Chinese Medicine, Zhengzhou, 450056 Henan China; 2 Department of Pharmacy, Henan University of Chinese Medicine, Zhengzhou, 450046 Henan China

**Keywords:** Extracellular vesicle, CRISPR/Cas system, Targeted delivery, Nanocarrier, Gene editing

## Abstract

Extracellular vesicles (EVs) have emerged as a promising platform for gene delivery owing to their natural properties and phenomenal functions, being able to circumvent the significant challenges associated with toxicity, problematic biocompatibility, and immunogenicity of the standard approaches. These features are of particularly interest for targeted delivery of the emerging clustered regularly interspaced short palindromic repeat (CRISPR)/CRISPR-associated (Cas) systems. However, the current efficiency of EV-meditated transport of CRISPR/Cas components remains insufficient due to numerous exogenous and endogenous barriers. Here, we comprehensively reviewed the current status of EV-based CRISPR/Cas delivery systems. In particular, we explored various strategies and methodologies available to potentially improve the loading capacity, safety, stability, targeting, and tracking for EV-based CRISPR/Cas system delivery. Additionally, we hypothesise the future avenues for the development of EV-based delivery systems that could pave the way for novel clinically valuable gene delivery approaches, and may potentially bridge the gap between gene editing technologies and the laboratory/clinical application of gene therapies.

**Graphical Abstract:**

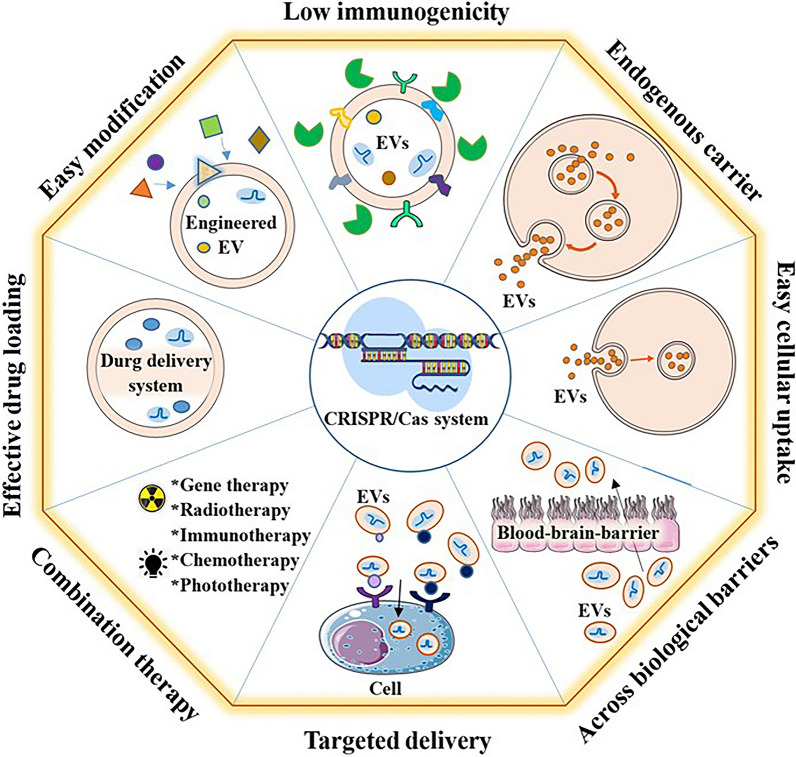

## Background

As a revolutionary genome editing tool, clustered regularly interspaced short palindromic repeat (CRISPR)/CRISPR-associated (Cas) genome editing systems have achieved rapid development and great success in the fields of biomedicine [1], agriculture [2], and manufacturing [3]. Compared with traditional gene editing tools, such as zinc finger nucleases and transcription activator-like effect nuclease, CRISPR/Cas systems offer unique advantages, including targeted editing of multiple sites, rapid generation of mutants, and single-guide RNA (sgRNA) designability [3]. In particular, the CRISPR/Cas9 RNA-guided endonuclease system is a powerful emerging tool that is the most thoroughly studied and widely applied and has been successfully employed for gene knock-in, knock-out, repair, and transcriptional regulation [4]. Regardless of whether CRISPR/Cas forms plasmid DNA (pDNA), transcribed mRNA, or pre-assembled ribonucleoprotein (RNP) complexes, its components must be delivered to the target cells by carriers with specific features, including high safety, stability, efficiency, and nontoxicity profiles. However, as the leading tool for delivering CRISPR/Cas in vivo, viral vectors have limitations related to packaging constraints, immunogenicity, carcinogenesis, scale-up production, and the longevity of Cas expression. Nonviral carriers also face various challenges, including rapid clearance, problematic biocompatibility, toxicity/immunogenicity, and potential issues with therapeutic cargo release [5].

Nanosized extracellular vesicles (EVs), which can be categorised into exosomes (30–150 nm) and microvesicles (MVs; 50–1000 nm), are released by all cell types into the extracellular milieu under various physiological and pathological conditions. Exosomes comprise intraluminal vesicles formed via the invagination of multivesicular endosomes (MVEs) membranes and are released into the extracellular space upon fusion of MVEs with the cell membrane. In contrast, MVs are highly heterogeneous EVs that are characterised based on their origin and are secreted via outward budding of the plasma membrane [1–3]. At the cellular level, particles of different sizes elicit unique uptake mechanisms; that is, particles smaller than 100 nm can be taken up via clathrin- or caveolae-mediated endocytosis, whereas larger complexes may require macropinocytosis [4, 5]. Consequently, larger aggregates are commonly directed toward lysosomal degradation or membrane recycling, whereas smaller vesicles may achieve higher rates of effective intracellular delivery [6]. However, their overlapping size and biomolecular cargo can often hinder the efficient identification and isolation of specific EV types [2]. Due to the rapid evolution of EV nomenclature, herein, we apply the general term ‘EVs’ to describe all cell-derived nanoparticles (NPs). Biochemical and proteomic analyses have effectively characterised various proteins, nucleic acids, lipids, and other components carried by EVs [7]. These components execute diverse pathophysiological functions by regulating myriad cell signalling pathways [8]. Thus, EVs have been used as potential biomarkers and natural therapeutic agents for diseases. In recent years, EVs have become increasingly popular in the ever-expanding field of gene delivery.

**Fig. 1:**
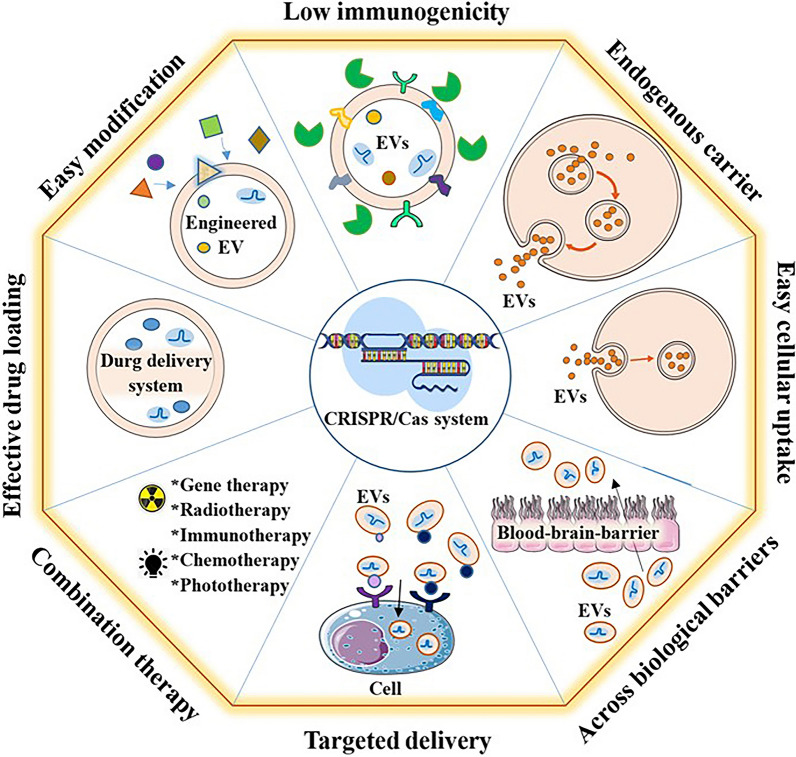
Advantages of extracellular vesicles (EVs)-based CRISPR/Cas systems delivery system

**Fig. 2:**
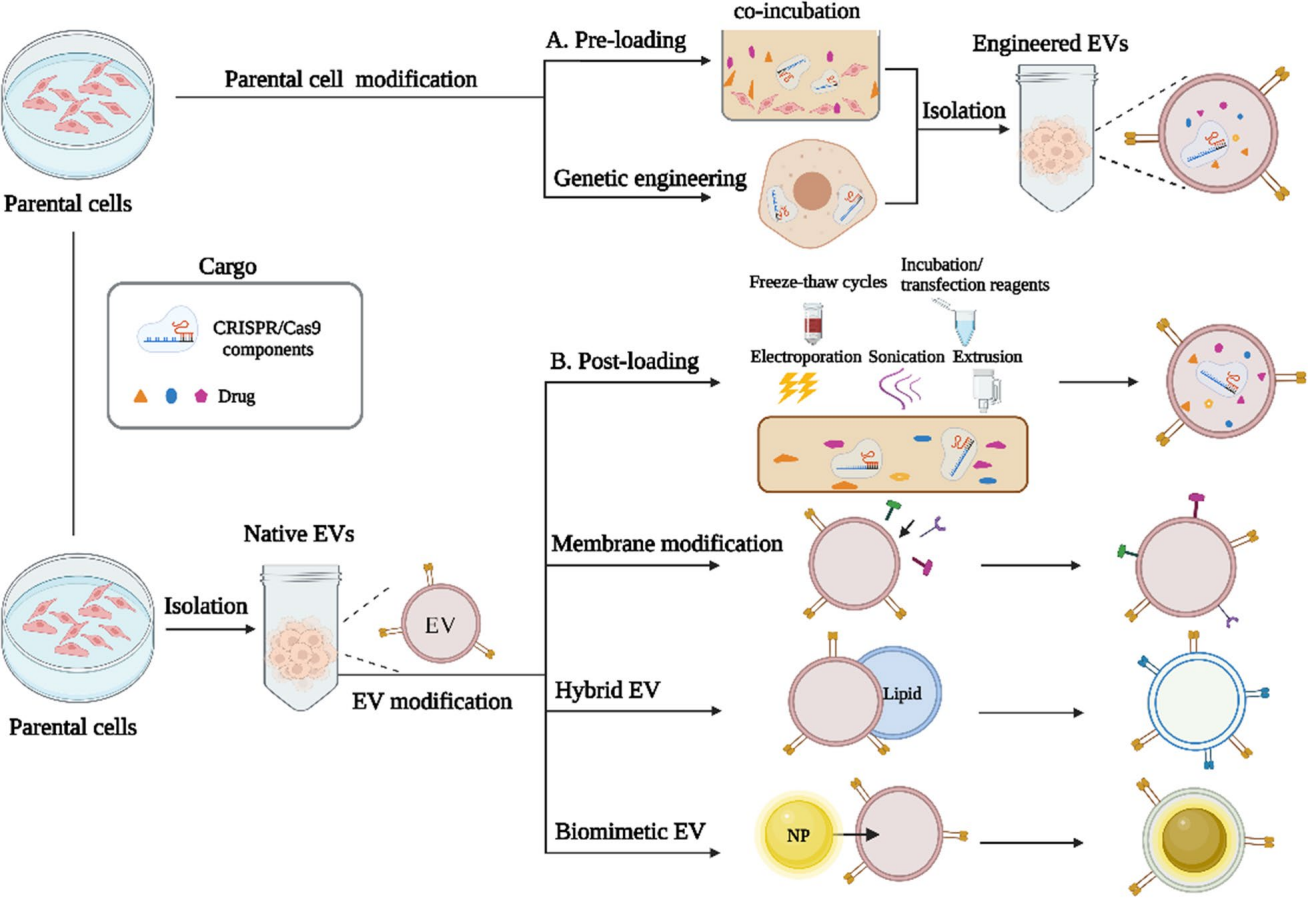
Modification strategies for the preparation of engineered EVs. EVs can be modified by indirect methods such as pre-loading cargo and genetic manipulation (**A**), or direct methods, including post-loading cargo (**B**), EV membrane modification and artificial EVs (such as hybrid EVs (HEs) and biomimetic EVs). As for cargo loading, there are three main approaches, including the pre-loading method, the post-loading method, and others. Created with Biorender.com

EVs represent a promising alternative delivery vector for the CRISPR/Cas9 components that circumvents the limitations of other carriers (Fig. 1). EVs have high biocompatibility and stability because they shield the cargo in phospholipid bilayer membranes or by expressing signalling molecules at high levels on their surfaces. Therefore, the coated cargo is not taken up allowing it to achieve a low clearance rate and long-term circulation [6–8]. Owing to their strong modifiability and efficient internalisation with few undesirable immune reactions, EVs are safe and feasible for the delivery of diverse cargoes [9, 10]. Indeed, targeting membrane-anchored ligands, or portions inherently expressed on the EVs surface, can achieve specificity [11]. When responding to internal or external stimuli, the intelligent release of EV cargo can be spatiotemporally controlled [12]. A wide range of cellular adhesion molecules facilitate EV penetration through biological barriers and migration into tissues or areas with no blood supply, thereby significantly improving the bioavailability and targeting of the EV cargo [13, 14]. Hence, natural EV carriers may represent an effective tool for the delivery of CRISPR/Cas9 components. Nevertheless, their transportation into target sites remains insufficient, owing to many exogenous and endogenous barriers. Many reviews have elucidated the design characteristics of each CRISPR/Cas system [15, 16], including the advantages, disadvantages, and unique physicochemical and physiological features. Hence, this review provides a comprehensive review of the current status of EV-based CRISPR/Cas delivery systems. Additionally, various emerging/prospective strategies and methodologies to improve the loading capacity, safety, stability, targeting, and tracking of design/modification/engineering of EVs to targeted delivery the CRISPR/Cas system, including pre-isolation or post-isolation modification and artificial EVs (Fig. 2). Finally, we dissect the current challenges and prospected future outlooks for EV-based CRISPR/Cas delivery to improve its editing efficacy and expand its practical applications in various fields.

## Loading

Generally, the loading approaches of active molecules in/on EVs can be divided into three categories, (1) pre-loading methods (cell-based loading, or the pre-isolation approach) involve loading cargo into EVs, which is then secreted in an EV-carrying manner from parent cells using transfection [17] or co-incubation [18] approaches. Although these methods are highly repeatable and relatively simple, the loading efficiency is typically low and highly dependent on the parent cell types, as well as cargo characteristics and concentration gradients (Fig. 2A). (2) Post-loading methods (direct loading, or post-isolation approach) harvest EVs from distinct sources and introduce cargo into EVs via co-incubation [9], electroporation [19], sonication, extrusion [20], transfection reagents [21], or freeze–thaw cycles [22] (Fig. 2B). This strategy is more customisable than the former and minimises the inclusion of other unnecessary substances. (3) Other loading methods include cellular nanoporation biochips [7], enveloped protein nanocages (EPNs), and artificial EVs.

### EV-loading pDNA

Among the three formats (pDNA, mRNA, or RNP), pDNA is the most widely used for CRISPR/Cas system delivery. Owing to their multi-drug loading capacity, biological barrier crossing, and targeting ability, EVs can be utilised as natural carriers for targeted CRISPR/Cas plasmid delivery, circumventing the underlying immunogenicity and toxicity of cationic materials [23]. However, repeated expression of Cas9 and sgRNA increases off-target risks and undesired mutations. Moreover, compression of more than 10 kb of pDNA into EVs, as well as its translocation to the nucleus, is difficult [24]. Nevertheless, various approaches, including transfection of donor cells [17], electroporation [19], and transfection kits [21], facilitate the loading of pDNA into EVs, which is then delivered to target cells. Table 1and Table 2 shows the outcomes and characteristics of each strategy for engineered EV-mediated delivery of CRISPR/Cas9 components. When the pDNA of CRISPR/dCas system be loaded into EVs, plasmid size or sequence determines the delivery efficiency of EVs to a certain extent [17, 25]. For instance, a delivery system, comprising engineered minicircle DNA loading EVs, that has greater efficiency in loading minicircle DNA and lead to significantly prolonged and higher transgene expression as compared to their parental plasmid counterparts [26, 27]. Because the high-speed centrifugation may destroy vesicles and reduce sample quality [21], the engineered EVs-mediated delivery of CRISPR/Cas9 imparted only a moderate editing efficiency (∼58% suppression) on the target cells, which requires separation and purification of EVs before and after transfection.

**Table 1:** Loading methods for EV-delivered CRISPR/Cas components

Loading approaches	Loading methods	EV sources	Load efficiency/ratio	Cargo	Advantages	Limitations	References
Pre-loading methods	Genetic engineering	Liver AML12 cells	20%–30%	pDNA	Simple, quick, common application	The off-target effect, limited load efficiency, potential carcinogenic risk	[17]
		HEK293T cells	/	pDNA			[177]
		HEK293T cells	22.3 ± 8.5 copies of Cas9 mRNA/100 EVs	Cas9 mRNA			[29]
		HEK293T cells	/	RNPs	High efficiency loading, high gene editing activity; versatile; intact structure	Difficulties in obtaining large quantities of EVs-RNPs	[63]
		HEK293T cells	50%–71%				[65]
		Retroviral virus	/				[61]
		HEK293T cells	~ 23%				[40]
		HEK293T cells	1.5–2%				[35]
		HEK293T cells, CHME-5 cells	< 1%				[36]
		Human iPSCs, HEK293T cells	7.9 RNPs/EV	Cas9 protein, sgRNA	High efficiency loading, high gene editing activity; versatile; quantitative measurements of transfer activity	Tedious preparation, time consuming, lack of stability and specific targeting	[23]
		HEK293T cells	/				[48]
		HeLa cells, HuH7 cells, Vero cells, CHO cells	10%				[37]
		U2OS cells, HEK293T cells	540 RNPs/EV				[47]
		HEK293T, HepAD38, HeLa, Huh7 cells	100 ng Cas9 protein/10 µg EVs				[39]
		Multiple cells	0.7%				[50]
		Expi293F cells	25 Cas9 molecules/EV				[46]
		All types of screened malignancy	/				[141]
	Co-incubation	Dendritic cells	/	pDNA	Intact structure, simple, and quick	Unknown long-term safety, low gene editing efficiency	[9]
Post-loading methods	Electroporation	HEK293T cells	/	pDNA	High efficiency loading, operation reproducibility, avoids the risk of gene transfer	EV damage, EV or RNA aggregation, not suitable for clinical application	[19]
		HEK293T cells, ovarian cancer cells	~ 2%	pDNA			[38]
		Hepatic stellate cells	20%	RNPs			[41]
		RBCs	18%	Cas9 mRNA			[30]
	Freeze–thaw, sonication, co-incubation	HEK293T cells	40%	pDNA	High efficiency loading, simple operation	EV integrity disruption and aggregation	[18]
		HEK293T cells	37.62%	RNPs			[22]
	Transfection kit	HEK293T cells	10 μg DNA/10^9^ EVs	pDNA	Straightforward, convenient, and cost-effective	Lower efficiency, potential toxicity	[21]
		Serum	/	RNPs			[42]
	Cell nanoporation	All cell types	/	Cas9 mRNA	High efficiency loading	Needing special equipment	[7]
	Co-incubation	MDA-MB-231 breast cancer cells	7.7 ng Cas9 protein/3.5 × 10^8^ EVs	Cas9 protein or sgRNA	Intact structure, simple, quick, and versatile operation	Low efficiency	[49]

**Table 2:** EV modifications for loading CRISPR/Cas components

Modification strategy of EVs	Loading mechanism	Disassembly mechanism	Model	References
Bioengineering	GFP-CD63 fused with Cas9-antiGFP nanobody	/	In vitro	[29, 177]
	CD63-com fused with ABP Com-sgRNA	/	In vivo	[40]
	ARRDC1 fused with WW-Cas9	/	In vivo	[47]
	CD9-HuR and ABE-AU-mRNA interaction	/	In vitro, in vivo	[29]
	VSV-G-assisted	Spontaneous release	In vivo, in vitro	[35, 50]
	split GFP complementation	/	In vitro, in vivo	[48]
	chemical-induced incorporation	/	In vitro, in vivo	[36]
	CD9-CIBN and Cas9-CRY2 interaction, light	Removal of the light	In vivo	[46]
Physical method (Freeze–thaw, sonication)	TDNs-EVs	/	In vitro, in vivo	[22]
Physical method (Freeze–thaw, co-incubation)	Multivalent electrostatic interaction	/	In vivo	[9, 18]

Hybrid EVs (HEs) with liposomes have offered a promising prospect for application in gene loading [9, 18]. More specifically, HEs are formed via freeze-thawing EVs and incubating them with a mixture of liposomes and plasmids for 12 h at 37 °C. As such, HEs are larger and more stable than EVs; however, they retain the same surface proteins, which can significantly enhance their loading, targeting efficacy, and cellular uptake efficiency, without affecting their native functions or integrity. Liposomes alone cannot effectively transfect or express encapsulated genes. Thus, the development of engineered EVs through nanobiotechnology holds promise for CRISPR/Cas delivery with the combined advantages of native EVs and synthetic NPs.

### EV-loading mRNA

Compared to the pDNA format, mRNAs have the advantages of a smaller molecular structure, rapid onset of action, and minimal off-target effects. Additionally, EV-based mRNA can provide high biostability and cellular uptake efficiency with negligible cytotoxicity. However, their application is limited by the fragility of the single-stranded structure Cas9 mRNA, which must be resolved for RNA protection during systemic shuttling. Direct encapsulation of donor cells represents the most common strategy for loading nucleic acids into EVs and is suitable for expressing high molecular weight molecules; however, this strategy has a relatively low loading efficiency [28]. To enhance RNA loading, the EVs membrane protein CD9 has been fused with human antigen R (HuR, RNA-binding protein) to generate an domain on the inner surface of EVs. In fact, addition of three AU-rich elements to the Cas9 mRNA sequence-rich element, CD9-HuR and AU facilitates the efficient loading of Cas9 mRNA that could be transfected into donor cells. Engineered CD9-HuR-EVs have considerable potential for ARE-modified Cas9 mRNA encapsulation [29]. One study reported that approximately 22.3 ± 8.5 copies of Cas9 mRNA were found in 100 engineered EVs, indicating a tenfold increase in loading efficiency.

Electroporation is another effective method for introducing Cas9 mRNA into EVs and utilises an external electric field to generate pores on the surface of EVs to transfer mRNA [6, 30]. Electroporated EVs have a higher antisense oligonucleotide delivery efficiency and lower toxicity than commercial transfection reagents. However, electroporation is not suitable for all RNA cargoes, such as modified microRNA and short hairpin RNA [31]. Electroporation can lead to EV aggregation, which decreases the loading efficiency and impairs their delivery properties. To mitigate this issue, membrane stabilisers, such as sucrose and trehalose, are used to maximise the colloidal stability of EVs and minimise the aggregation caused during electroporation [32, 33]. Hence, the electroporation conditions must be optimised on a case-by-case basis depending on the type of payload and EV source. Additionally, other post-loading approaches, such as sonication, extrusion [20], freeze-thawing [18], and chemical transfection [34], are used to incorporate RNA into EVs. Despite their simplicity and ease of implementation, these methods share the limitation of poor cargo capacity, particularly for encapsulating hydrophilic molecules. This issue may be due to EV size, zeta potential, or polydispersity index, which could subsequently alter the pharmacokinetic properties of EVs. To overcome this issue, a highly-efficient cargo loading method designated ‘sonication and extrusion-assisted active loading’ (SEAL) has been developed. Through the combination of sonication and extrusion, SEAL has exhibited an approximately tenfold enhancement of drug encapsulation efficiency in case of doxorubicin-loaded EVs [20]. Additionally, a cellular nanoporation biochip allows plasmids to shuttle from the buffer to source cells through a series of electric pulses [7]. Compared to conventional methods, this strategy provides a 50-fold increase in EV yield and a 1000-fold increase in the loading of intact mRNA transcripts. Given that nanoporation-stimulated EVs are highly independent of source cells and transfected vectors, minimal cell death or activation of apoptotic pathways occurs in parental cells.

### EV-loading RNPs

As the most straightforward and efficient format, functional ribonucleoprotein complexes (RNPs) are localised inside the nucleus of host cells through minimal intracellular processing. Based on the RNP format, the delicate sgRNA molecules are significantly protected from degradation, and the occurrence of off-target mutations is reduced. As RNP delivery cannot trigger a cellular immune response and also offers control over stoichiometry, it helps set up dosage parameters in disease therapy [5, 24]. By genetic engineering, RNPs can be co-packaged into EVs without requiring additional modification of the producer cells [35–40]. However, the ability of non-transformed cell lines to package CRISPR/Cas components is unknown, and this method is limited by its cytotoxicity, poor specificity, and low load efficiency.

Optimising this platform with engineered EVs may be an effective means of improving the loading efficiency of RNPs. For instance, SpCas9/sgRNA RNPs have been shown to be selectively and actively packaged into engineered virus-like particles (eVLPs) to enable exon skipping in muscular cells. In every endogenous genomic locus test, the results have confirmed that EV-loaded RNPs outperform pDNA transfection with 32.5–50% indels and an exon skipping efficiency of over 90% [23]. However, those engineered EVs are more likely to be VLPs than typical EVs, which may directly affect the approach of gene editing. In view of the similarity between EVs and VLPs, the viral proteins, such as vesicular stomatitis virus glycoprotein (VSV-G), were incorporated into EVs that exploits the tissue targeting advantages of viral delivery, but avoid the risks associated with viral genome integration and prolonged-expression of the editing component [35, 36]. Rather than using adeno-associated viruses or tumour cell-derived EVs, loading RNPs into hepatic stellate cells [41] and serum-derived [42] EVs with electroporation and protein transfectants will enable the rapid and safe delivery of CRISPR/Cas components.

To further improve load efficiency, EV-loading RNP leverages the high binding affinity between green fluorescent protein (GFP) and GFP nanobodies [43] or the specific interaction of RNA aptamers and aptamer-binding proteins (ABP) [44]. The former RNPs into engineered EVs by fusing GFP to EVs enriched protein CD63, and fusing single-chain GFP-binding antibody to Cas9 [43]. While in the latter, CD63 was modified by appending the aptamer-binding protein Com to both the N- and C-termini of the protein and replaced stem loop 2 of the sgRNA with aptameric RNA com. Com-com interaction allows for the recruitment of Cas9 to EVs via sgRNA. This system demonstrated a 2 to 5 times more efficient recruitment of Cas9 with com relative to spontaneous loading and co-packaged RNPs showed 10 times higher than the combination of the individually packaged RNPs for multiplex genome editing [40]. These features make engineered EVs an ideal delivery tool for multiplex genome editing. Cas9 RNPs can be efficiently loaded into tetrahedral DNA nanostructures (TDNs) engineered EVs with a tenfold improvement of binding affinity. Meanwhile, sonication or freeze–thaw cycles increased the loading efficiency of RNPs into TDNs (15.34% vs. 37.62%) [22]. In addition, enhancing RNP escape, optimising RNA copy numbers, and selecting appropriate EV source cells may improve the delivery efficiency of engineered EV-based CRISPR/Cas systems [40].

### EV-loading Cas9 protein and sgRNA alone

Packaging Cas9 protein into EVs through transfected donor cells is often inefficient, cumbersome, time-consuming, and incompatible with certain EV sources, such as human plasma and bovine milk [37, 39]. Several attempts have been made to load specific target proteins into EVs, including the fusion of target proteins with a constitutive EV protein [45–48], reversible protein–protein interactions [49, 50], fusion of the EV peptide [51], and EPNs [52, 53]. Through the interaction between the WW domain (~ 40 amino acids each) of neural precursor cell-expressed developmentally downregulated gene 4 family proteins and arrestin domain-containing protein 1(ARRDC1), WW domain modified-Cas9 and sgRNA were co-transfected into cells and then WW-Cas9-sgRNA were loaded into EVs [47]. In a similar way, signal peptides were selected to construct the optimized editing system CRISPR/Cas13d plasmids and engineered EVs were extracted from HEK293T cells, the Cas13d‐gRNA complex delivered by EVs successfully disrupted the RNA of both exogenous and endogenous genes in a short‐acting manner and ultimately reduced the expression of target proteins [51]. However, these strategies do not provide a clear releasing mechanism for editing complexes in the target cells. Thus, the regulation of protein–protein interactions seems more attractive. Through the fusion of Cas9 with EV transmembrane fluorescent protein, induced reversible hetero-dimerisation results in efficient loading with aCas9 into EV [36]. By contrast, through optically reversible protein–protein interactions, Cas9 have been loaded into EVs through the interaction of CD9-cryptochrome 2 (CRY2) and CRY-interacting basic-helix-loop-helix 1 (CIBN) when activated by light. The chemical dimerization system performed a less efficiency than that of the CIBN-CRY2 photosystem [46]. Furthermore, the fusion of octapeptide onto the N-terminus of Cas9 proteins promotes Cas9 myristoylation and encapsulation into vesicular stomatitis VSV-G-modified EVs, which increases the transfection potential with encapsulated Cas9 accounting for 0.7% of total EVs [50]. However, more in-depth studies on the distribution and homing of designed EVs in vivo are needed [48].

Alternatively, passive incubation-specific modification of Cas9 provides a quick, versatile, and simple method for loading proteins into EVs [49]. After Cas9 proteins are bound to cationic lipids, they can be further complexed with EVs via passive incubation. The resulting EVs retain native features following protein-loading and deliver Cas9 proteins with an efficiency similar to that of commercial transfection reagents; however, they exhibit less toxicity while outperforming electroporation [54]. In addition, the EPN method has been reported to significantly increase EV-loading capacity, in which each EPN can be actively loaded with up to 60 cargo molecules. The EPN method demonstrated the significantly potential for Cas protein packaging in EVs [52, 53]. This strategy provides easy-to-modulate and low-cost machinery for rapidly enveloping protein cargo into engineered EVs and their precise delivery to target cells.

Another fundamental component in the RNA-based strategy is sgRNA, which can be generated either by in vitro transcribed (IVT) or solid-phase synthesis. IVT sgRNA can induce immune responses and cause cell apoptosis [55, 56], whereas chemically synthesized sgRNA cannot, due to the lack of a 5′-triphosphate group. During sgRNA synthesis, the structurally well-defined assembly enables high homogeneity and site-specific incorporation of modified sgRNA [57]. For example, synthetic sgRNA was flanked by self-cleaving ribozymes hammerhead and hepatitis D virus ribozyme, resulting in the sgRNA being selectively and actively packaged into EVs with at least a fourfold improvement [23]. Chemically modified sgRNAs can be developed to enhance enzymatic stability, editing efficiency, and specificity, and to reduce immunogenicity and off-target effects, such as 2′-O-methyl-PS, 2′-O-methyl-3′-thiophosphonoacetate [58], and modified crRNA/tracrRNA nucleotides [59]. Additionally, other factors that determine the loading efficiency of EVs require thorough investigation, including the kinetics of release, biodistribution, clearance, and intracellular fate after EV internalisation [54].

Although many methods for EV-mediated transfer of CRISPR-Cas systems have been established, there are still many challenges in delivering the functional CRISPR-Cas systems into the nucleus. Due to the scarcity of existing literature to the EVs load efficiency, it presents a challenge to conduct a comprehensive and systematic comparison of diverse loading methods. Hence, a thorough exploration of the release rate of EVs-loaded cargo and their translation efficiency is imperative. Next, the ambiguity of EVs definition has limited their application since the lack of unique markers to define specific subtypes in various secreted vesicles [60, 61]. It is urgent to develop a approach to distinguish or isolate specific types of vesicles [62]. Moreover, the equimolar concentration between Cas proteins and sgRNA can maximize the formation of their conjugates. But their accurate quantitation in the cytoplasm is very difficult to conduct [63, 64]. Except that, due to relies on passive diffusion or complexation of the molecule with a cell or organelle, pre-loading methods may rely on a number of factors such as pondus hydrogenii (pH), osmotic pressure, and electric charge or hydrophobicity. Therefore, more efforts are needed to optimize the existing techniques or to develop new way in the future.

## Safety

Before being used to deliver CRISPR/Cas9 components, EV biosafety and the associated adverse effects must be evaluated to ensure that EVs do not have deleterious roles (as outlined in the limitations section of Table2) [9, 18, 38, 65]. EV characteristics inherently depend on donor cells; therefore, choosing the proper cell source, such as autologous or non-autologous sources, is critical for safe and effective EV-based delivery. An autologous source presents the same host cells and guarantees ideal materials while avoiding mismatched antigens and the risk of host immune responses [66]. However, EV preparation from autologous sources offers challenging and time-sensitive availability. By establishing homologous/identical donor cell banks, large-scale reserves can be obtained conveniently and promptly, for dendritic cells (DCs) [67], serum [42], and RBC-derived EVs [30]. Non-autologous sources are preferred due to the regulatory/commercial desirability of a streamlined, exceptionally well-qualified product. Indeed, non-autologous sources have been used as safe, economic, and practical sources for EV production [68, 69]. Selecting appropriate major histocompatibility complex (MHC) cell sources will reduce or prevent unnecessary immunogenicity. The desired cargo and molecular attributes must also be selected before EV preparation [54].

Several modification strategies have been proposed for reducing the immunogenicity of EVs. To further improve delivery and safety, a strategy combining engineered EVs with antibodies, liposomes, or EPNs has been adopted [52, 70, 71]. For example, chimeric protein (pre-miR-199a-3p)-modified EVs display low immunogenicity and minimal evidence of changes in immune markers, thus, expanding the application scope of EVs in cargo delivery [66]. EV immunogenicity has excellent potential for exploring novel vaccines or vaccine vectors. Through gene and chemical modifications, bacterial membrane EVs can be endowed with more functions, which can be used in immunotherapy for infectious and non-infectious diseases [72]. Additionally, engineered EVs can be used for anti-infection [73] and anti-systemic inflammation [9] purposes. For instance, by fusing ACE2 or chondrocyte-affinity peptides, engineered EVs can inhibit viral infection [73] or attenuate the progression of osteoarthritis [9]. Decreasing the immunogenicity of CRISPR/Cas components by deleting specific genes encoding unwanted immunogenic proteins [74], removing Cas9 epitopes [75], and using suitable Cas proteins or orthologues [76], is another effective strategy for ensuring safe and effective targeted delivery.

## Stability

To deliver the gene/genome-editing component to the target site, it is crucial to safeguard it from degradation or neutralization by the complex physiological microenvironment in vivo, which includes mechanical, biological, and immunologic barriers [77]. However, exogenous EVs can be rapidly cleared owing to macrophage engulfing associated with the reticuloendothelial system (RES) [78]. Avoiding the RES effect is an effective strategy for increasing EVs' stability and accumulation in target sites [79]. When EVs are ≤ 100 nm, their stability in the target tissue can be enhanced by improving the permeability and retention effect [78, 80, 81]. In addition, by decreasing/masking prophagocytic molecules or introducing anti-phagocytic signals on EV surfaces, such as polyethylene glycol (PEG), CD47 protein, glycans, phosphatidylserine (PS), and lipid fusion, multiple strategies have been developed to reduce non-specific phagocytic uptake.

### Stability modification of EV membranes

**Fig. 3:**
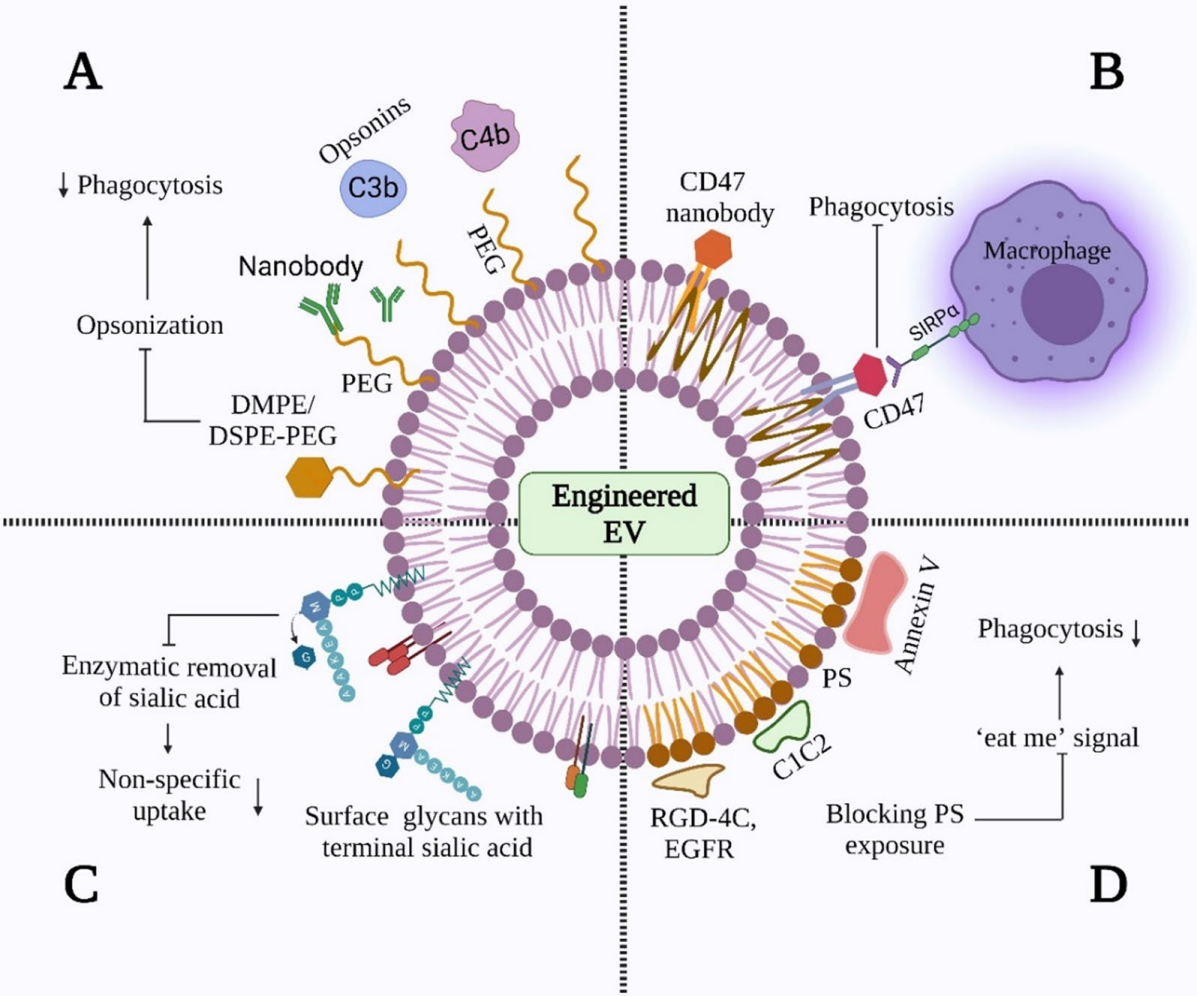
Diverse approaches for enhancing EV stability. **A** PEGylated EVs. The EV surface was modified with PEG by inserting the nanobodies. Distearoyl phosphatidyl ethanolamine (DSPE), dimyristoyl phosphatidyl ethanolamine (DMPE), and PEGylated EVs prevent complement system-mediated opsonisation and decrease phagocytosis. **B** CD47 mediation. **C** Removal of sialic acid. The sialic acid residues of EV surface glycans were removed via neuraminidase, reducing their non-specific uptake, and thereby altering their biodistribution. **D** Blocking phosphatidylserine (PS). By inhibiting ‘eat me’ signals with PS-binding molecules, such as RGD-4C, epidermal growth factor receptor (EGFR), Annexin V, and C1C2 domain of lactadherin, then PS was blocked, which subsequently decreased phagocytosis. Created with Biorender.com

*PEG:* PEG is a commonly used polymer for improving the stealth performance of EV delivery systems. By forming a hydration layer around EVs, PEG sterically hinders the interaction of EVs with opsonins and decreases the recognition of macrophages (Fig. 3A). With modifications of the nanobody conjugate [82] or lipid anchor [83], PEGylated EVs can escape the RES and prolong EV circulation time. PEGylated macrophage-derived EVs enhance tumor tissue specific uptake and prolong circulation time by sevenfold higher without altering their morphology, size, or protein composition, and the classical biodistribution pattern of EVs. Nevertheless, owing to the immunoglobulin (Ig)M antibodies raised against PEG, the clearance of PEGylated EVs is highly accelerated and may occur at repeated dosing [84]. Therefore, other polymers or biomaterials can replace PEG to modify EVs, such as hyperbranched polymers, polymeric fibrous scaffolds, or cholesterol [85, 86].

*CD47 protein:* CD47 is the most common molecule employed to modulate EV stability and biodistribution. The interaction between CD47 and the signal regulatory protein α (SIRPα) expressed by macrophages allows the latter to identify CD47-SIRPα as self-substances that inhibit their activation and activates the ‘don’t eat me’ signal [87] (Fig. 3B). The CD47 on the EV surface can contribute to the clearance suppression of EVs and enhanced delivery efficiency. For instance, by inserting the glioma targeting peptides into the N-terminus of CD47, the accumulation of engineered EVs was increased by 1–1.5 times, and the survival time of mice was prolonged to 45–49 days [7]. However, owing to the limitations of genetic engineering methods, CD47 modification is not feasible for all parental cell types. Alternatively, using artificial EV-loaded anti-phagocytic signals [88], CD47 nanobody [89, 90], or an enzymatically ligated mimicking CD47 peptide modified onto EV surfaces appears to be a promising approach [91]. In addition to CD47, exploring new molecules, such as CD55, CD59, CD31, and CD24, which can further enhance its stability, is necessary [92].

*Glycans:* Glycans are crucial cell communication intermediaries and potential novel indicators of EV heterogeneity [93]. By inducing an N-glycosylation sequence to the Lamp2b N-terminus of EVs, engineered EVs can protect modified peptides from degradation and, thus, improve their stability [92]. Generally, hydrophilic NPs are more challenging to recognise by phagocytes than hydrophobic NPs. Therefore, they can replace hydrophobic glycans, such as polysorbate 80, surfactants, block polymers, and gangliosides, to avoid RES phagocytosis and enhance the stability of modified EVs [86, 94]. Glycan-engineered EVs appear to be essential for recognition and uptake by recipient cells [93]. For example, modification by glycans or glycosylated phospholipids reduces the non-targeting of EVs to the liver, with a more significant proportion of vesicles taken up by target organs [95, 96]. In addition, the enzymatic removal of sialic acids and insertion of palmitoyl-Le^Y^ into glioblastoma EVs led to a fourfold increase in uptake, leading to an increase in CD8^+^ and CD4^+^ T cell responses [97] (Fig. 3C). As the glycocalyx composition of EVs is a fundamental factor in DC uptake, glycocalyx potentiates of EVs could be applied as an anticancer vaccine in immune-related therapies. However, its capacity to stimulate the immune system requires further investigation.

*PS:* PS is located in the inner leaflet of the cellular membrane. When relocated to the outer leaflet of the cellular membrane, PS serves as an ‘eat me’ signal that facilitates the recognition and engulfment of macrophages [98]. Given that PS enrichment in EVs may expose the outer leaflet during immune and blood coagulation processes [99], PS-displayed EVs are readily eliminated from the bloodstream. Therefore, blocking PS exposure on EV surfaces is a promising strategy for reducing the clearance rate of EVs and enhancing their circulation kinetics. For example, the PS moieties can be blocked with annexin V [100] and lactadherin (C1C2) domains, or with arginine-glycine-aspartic acid (RGD)-4C peptide [101], peptide nanobodies against the epidermal growth factor receptor (EGFR) [102] and C1C2 domain. These strategies reduce unnecessary macrophage engulfment and confer a targeting capability to modified EVs (Fig. 3D). The long-term effectiveness of PS and glycans under dynamic conditions in vivo requires further investigation.

**Fig. 4:**
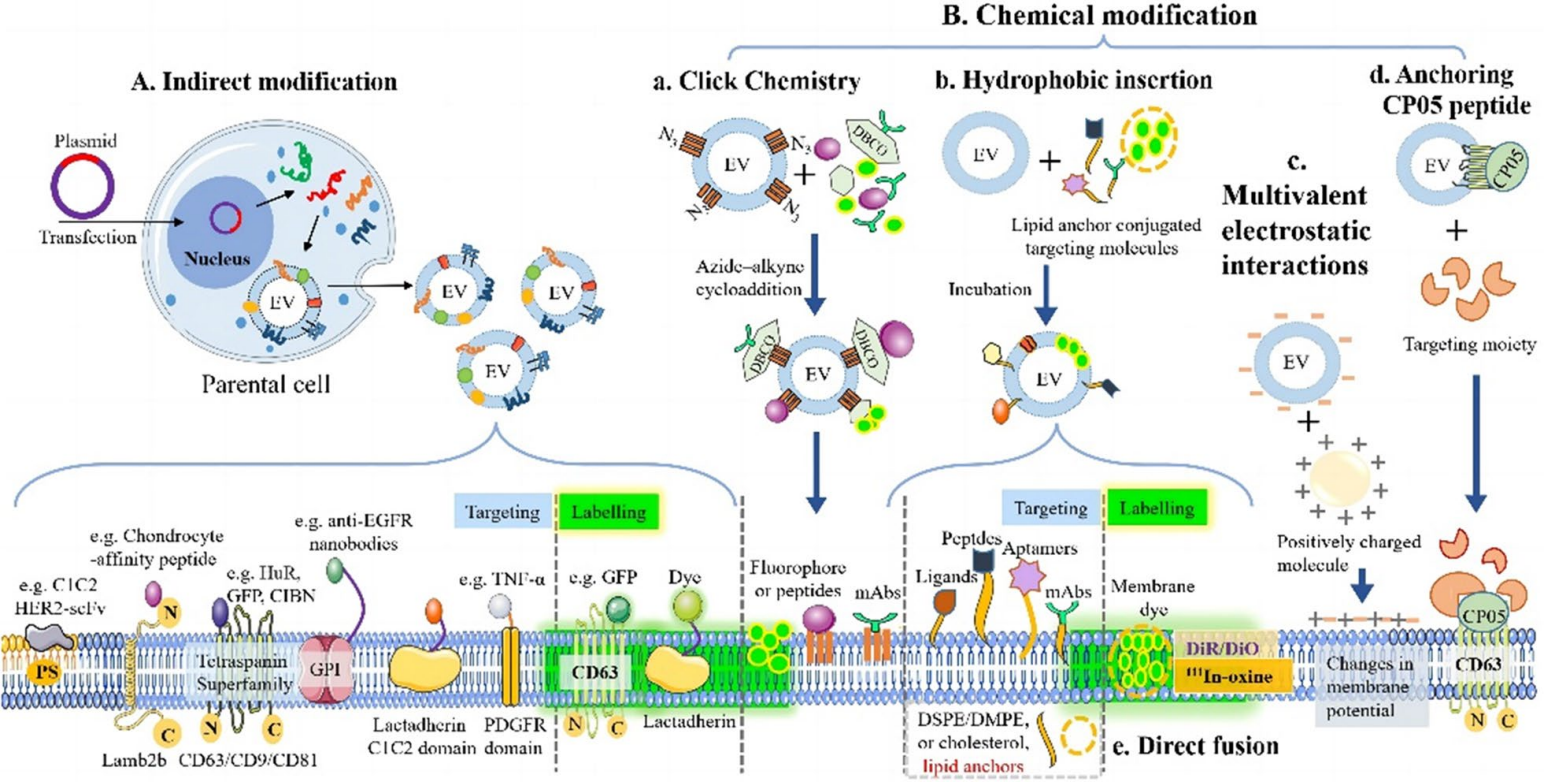
Different modification strategies for improving EV targeting. **A** Indirect modification. Overexpressed targeting molecules in parental cells fused to EV-enriched fusion partner proteins by vesiculation to obtain EV targeting. **B** Chemical modification. a. Click chemistry. Through azide–alkyne cycloaddition, different biomolecules are covalently bound to EV surfaces that endow them with specific functionality. b. Lipid/hydrophilic insertion. Lipids or hydrophilic molecules can be inserted into the lipid bilayer of EVs and were displayed on the EV surfaces. c. Multivalent electrostatic interactions. Change the membrane potential by binding to cations. d. CP05 peptide anchor. Based on the high affinity of CP05 peptide for CD63 molecule, the targeting moiety was loaded on the EV surface. e. Direct fusion. With the help of lipophilic nature, markers (such as DiR/DiO, 111In-oxine) are directly fused with the lipid bilayer of EVs. **C** Physical modification. Under the guidance of MF, EVs can be magnetically navigated to target sites

**Fig. 5:**
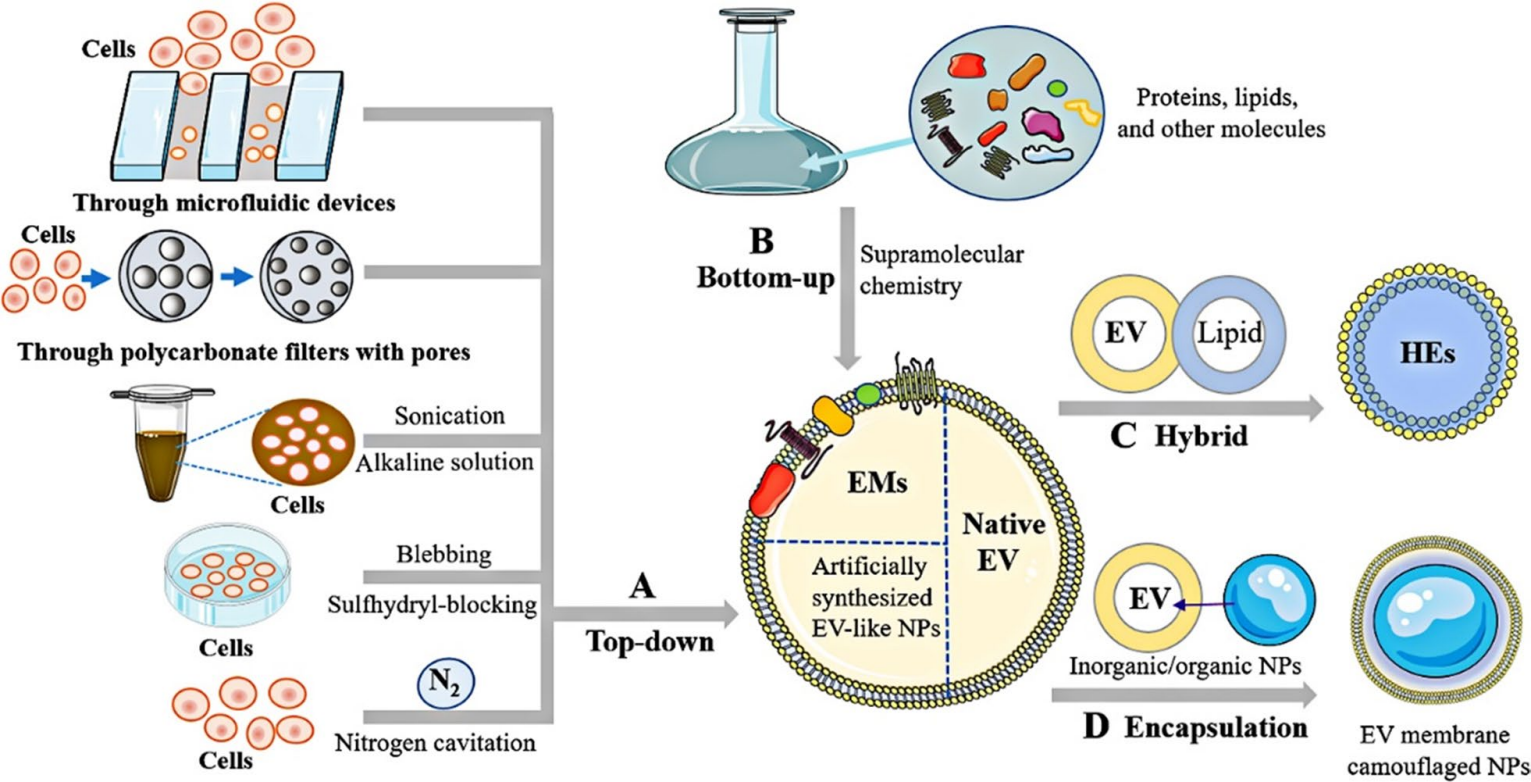
Different strategies for preparing artificial EVs. **A** Top-down strategy. Artificial EVs were produced with the following approaches: cells were forced to pass through membrane pores or microfluidic devices; cells were exposed to sonication in alkaline solution; cells were disrupted by nitrogen cavitation; cells released EVs by cell membrane blebbing with sulfhydryl-blocking. **B** Bottom-up strategy. By supramolecular chemistry, synthetic materials combined fundamental components from cells to form the EMs. **C** Hybrid strategy. The native EVs hybridised with synthetic liposome NPs/other sources of EVs to form the HEs. **D** Encapsulation strategy. The inorganic or organic NPs were encapsulated with the native EV membrane that obtained EV membrane-camouflaged NPs

*Lipid fusion:* Considering that different ‘stealthy’ surface molecules might be beneficial to improve EV stability, many strategies for EV modification have been developed to improve their stability. For instance, EVs have been fused with lipids, exogenous membrane proteins, and polylactic-co-glycolic acid (PLGA) NPs [86] (Figs. 4C and 5C-D). As shown in Fig. 2, with induction by freeze freeze–thaw cycles, electrostatic interactions, or co-incubation, HEs are expected to exhibit effective loading and improved stability. This may be caused by EVs escaping from the endosome trap, thereby increasing CRISPR/Cas9 cargo availability [9, 18, 49]. Exogenous membrane proteins can also be introduced into EVs to optimise their surface characteristics, thereby reducing their immunogenicity and prolonging their half-life [103]. Similarly, EV properties can be modified by inserting more liposomes embedded with peptides or 'do not eat me' molecules, such as CD47, PD-L1, and CD24. Given that EVs confer immune-evasive properties [104], the uptake of hybridised EV-PLGA NPs by macrophages and the immune response are significantly reduced, while their circulating half-life increases by approximately 3.5-fold [105].

### Endolysosomal escape

After CRISPR vectors enter the lysosomes via receptor-mediated endocytosis, digestive enzymes and low pondus hydrogenii (pH) environments can destroy the CRISPR/Cas9 cargo [106]. Thus, many strategies have been developed to bypass the endolysosomal pathway and successfully deliver therapeutic genes or drugs. These strategies include the introduction of specific molecules onto the EV surface [107, 108], combining EVs with compounds [109], and introducing pH-sensitive constituents [105]. Once specific molecules, such as fusogenic peptides [110] and cell-penetrating peptides [111], are introduced onto the surface of EVs, they can either be covalently conjugated with the Cas9 protein [112] or RNPs via ionic interactions [113] to facilitate the effective release of cargo and tropism. Moreover, the combination of EVs with autophagy inhibitors could effectively improve the stability and gene editing efficiency of the EV-loaded CRISPR/Cas9 system [114]. Inclusion of specific agents, such as chloroquine, amantadine, and bafilomycin A, can block acidification of the endosomal compartment, leading to swelling and bursting of endosomes [109]. Hence, pH-sensitive constituents, such as PLGA, that are introduced into EVs, are essential for the protection of RNA, preventing its degradation, and enabling cellular delivery through intracellular release [105, 115]. Finally, toxins escaping the degradative route can be engineered into EV membranes to enhance endolysosomal escape and cargo release. However, the lack of a feasible bioassay has hampered our understanding of the biological processes underlying EV uptake and cargo delivery to recipient cells. Therefore, an EV-mediated tetraspanin-tTA delivery assay was developed, which may improve our current understanding regarding the mechanisms of cytoplasmic cargo delivery in electric vehicles [116].

### EV storage

During the preparation of EVs, it is particularly important for their functionality that the purified EVs can be stored stably more completely. Increasing evidence suggests that EV concentration, physical properties, and functionality are affected by long-term storage. Among the storage conditions, temperature proved to be the most critical factor, and EVs were usually stored at 4, − 20, or − 80 °C [117]. The storage period determines the best storage temperatures for EVs; isolated EVs can be maintained at 4 °C for days or weeks and at − 80 °C for months [118]. Despite being an alternative for long-term storage, lyophilisation affects vesicle integrity during reconstitution. Thus, phosphate buffer solution supplemented with human albumin and trehalose added to the storage medium is recommended. Moreover, human albumin and trehalose significantly improved the recovery rate of EVs when the EV samples were diluted [119]. Freeze–thaw cycles can significantly affect EV durability, owing to the vulnerability of their PS moieties. Therefore, according to downstream analyses and experimental settings, EVs should be processed from off-the-shelf fresh, non-archival samples [120].

## Targeting

In addition to their high stability and safety, EV targeting is crucial for the precise delivery of CRISPR/Cas9 components to the target sites. Off-site delivery may lead to mutations in offspring or new disease development, which may raise severe ethical concerns [121]. Based on the intrinsic targeting properties and cell tropism of EVs [31, 122], specific cell-derived EVs provide potential vehicles for CRISPR/Cas9 targeted delivery in vivo [123]. Nevertheless, the properties of EVs alone are insufficient to guarantee selective delivery [38]. Various strategies for designing EV structures have been developed, including indirect or direct modification (Fig. 4) and artificial EVs (Fig. 5), to ensure EV accumulation in target tissues or organs. Indirect modification of EVs is performed by transfection of parental cells, and direct modification is realised through physical and chemical modification. Artificial EVs can be fabricated using top-down, bottom-up, biohybrid, or membrane-coated technologies. Table 3 outlines relevant studies that have utilised targeted molecules and their derivatives for specifically directing EVs to target cells for disease treatment. As described below, these approaches could lead to higher drug accumulation in target cells, reducing off-target effects and improving their efficacy.

**Table 3:** Modification methods for EVs targeted delivery

Approaches	Methods	Targeting molecules	Cargo	Target cells	References
Genetic engineering	Conjugated with Lamp2b	Chondrocyte-affinity peptide	pDNA	Chondrocyte	[9]
	Conjugated with CD63	GFP	RNP	Target tissues for enrichment of EVs	[177]
	Conjugated with CD9	HuR	AntimiR-155 or CRISPR/dCas9	Liver injury cells	[29]
		CIBN-CRY2	Cas9	HEK293, HepG2 cells	[46]
	PDGFR	TNF-α ligand	Cas9	Solid cancers expressing TNF receptor	[141]
	Chimeric-antigen receptor	Antigen	sgRNA/Cas9 plasmids	B-cell malignancies	[19]
	Signal peptide	Inflammatory factors	CRISPR/CasRx	Acute inflammatory tissues	[51]
Physical engineering	Magnetic field	Iron oxide NPs	Therapeutic molecules	Magnetic field position, endothelial cells and neurons	[145]
	Ultrasound-targeted microbubble destruction	Tissue-specific microRNA, fat brown transcription factor: PGC1α	RNP	Ultrasound position, dermal papilla cells	[149]
Chemical modification	Multivalent electrostatic interaction	Breast cancer-targeted biological molecules	Cationic lipid-Cas9 protein	Breast cancer cells	[49]
	Aptamer incorporation	Cholesterol anchoring-TDNs	RNPs	HepG2 cells, liver cancer cells; xenograft tumour models	[22]
	Lipid/hydrophobic insertion	Cell targeted biological molecules	pDNA, Cas9 mRNA	Cancer cells, chondrocyte	[9, 18, 49]

### Indirect modification

By engineering the parental cells with plasmid vectors expressing targeting moieties, such as peptides and receptors, the targeting moieties are displayed on the outer surface of the EVs to enhance their targeting ability, high stability, specificity, and increased safety **(**Fig. 4A). Common transmembrane proteins include the lysosome-associated membrane protein 2B (LAMP-2B) [124], tetraspanins [29, 125, 126], glycosyl-phosphatidyl-inositol (GPI) [127], C1C2 [128], and platelet-derived growth factor receptors (PDGFR) [31, 129], among others (Fig. 4A**)**. LAMP-2B is the most widely used EV surface protein for displaying a targeting motif, i.e., cell-specific binding peptides or antibodies targeting specific organs or tissues can be genetically modified at the N- [130] or C-terminus [131] of LAMP-2B to realise their targeting effects. For instance, by conjugation with LAMP-2B, the amino-acid sequence CGNKRTR (tLyp-1 peptide) becomes expressed on the EV surface for targeted delivery, resulting in a two-fold increase in the delivery of tLYP-1-labelled EVs to human lung cancer cells [132]. However, peptide degradation by endosomal proteases makes it challenging to obtain the desired yield of peptide-functionalised EVs. To prevent this degradation, two strategies have been suggested that confer high resistance to protease activity: that is, target peptides with a glycosylation sequence [133] and utilisation of D-isomers [134]. Compared with peptides, the antibodies, ankyrin repeat proteins that target HER2 fused with LAMP-2B exhibit higher binding affinities toward receptors on HER2-positive breast cancers cells, showing a four-fold uptake of engineered EVs in vitro [135].

As a viable and straightforward strategy, the tetraspanin superfamily (CD63/CD9/CD81) is often selected for EV modification with target molecules [29, 125, 136]. By conjugating CD63 with apoprotein-A1 and further bonding to scavenger receptors expressed on hepatocellular carcinoma cells, engineered EVs showed a two-fold increase in uptake for targeted delivery [136]. By sequential deletions of CD63's transmembrane helix, it establishs a novel and topologically distinct scaffolds that allow for flexible engineering of EV surface, thus facilitating targeted cargo delivery and adhesion [137]. Nevertheless, whether overexpressed tetraspanins affect producer cells or EV composition is unclear. In addition, other transmembrane proteins can be used as EV membranes to increase EV targeting, such as GPI [138], C1C2 [102, 139], and PDGFR [140, 141]*. I*ndeed, new receptors have been identified for precise delivery to target organs or cells (Table 3). For example, electroporation-loading of MYC oncogene-targeting sgRNA/Cas9 plasmids into CD19-chimeric-antigen receptor-modified HEK293T cells produced EVs results in a higher distribution in CD19 positive tumour tissues and more effective MYC genome editing than that of plain EVs [19]. Hence, expressing specific targeting moieties on EV surfaces by genetic engineering could serve as an effective strategy for active targeted delivery, thereby facilitating future EV-based therapeutic development.

### Direct modification

When the source cells for EVs are not easily modifiable, direct modification of EVs offers a more reliable alternative [142], direct modification, including physical or chemical modification, or the use of artificial EVs, can prove more reliable and promising compared to the indirect modification. As physical modification has the potential to damage EVs, surface labelling of EVs with targeting moieties is primarily implemented by chemical modification, i.e., covalent and non-covalent attachments. Compared to gene engineering of parent cells, chemical modifications present several advantages, including faster reaction time, high specificity, and compatibility with organic and aqueous buffers [143, 144].

#### Physical modification

Apart from the membrane loading of target molecules, many methods have been explored to load functional nanomaterials into EV with minimal damage to EVs and their functions, such as co-incubation, ultrasound, and electroporation [12]. Given that, physical exogenous guidance can lead to the localisation of EV-based delivery systems. Remote navigation by external fields, such as magnetic field (MF) [145] (Fig. 4C) and ultrasound [146], is directed to the precise location of target sites and leads to an increase in drug release in situ. Engineered EVs with potential magnetic targeting in vivo have been obtained by incubating superparamagnetic or iron oxide nanoparticles with MSCs. Under the guidance of an external MF, EVs can be magnetically navigated to target sites, with a precision 1.7–5.1 times higher than that of the control group [145], and drug release is controlled in time and space [147]. Ultrasound is another strategy for physical navigation widely employed in medicine for diagnostic and therapeutic applications. Although it cannot be used for external navigation of delivery systems, ultrasound can improve the site-specific infiltration of systemically administered drugs and trigger their release from responsive delivery carriers. Under conditions of local ultrasound exposure, EV-based nanosonosensitiser and mRNA delivery systems show targeted accumulation with minimised off-target effects [146, 148]. In order to enhance delivery efficiency and biocompatibility, a novel engineered EVs have been synthesized by microbubble-nanoliposomal. By utilizing microbubble cavitation-induced sonoporation, the RNPs were effectively delivered by engineered EVs and the target genes were edited both in vitro and in vivo with high efficiency [149].

Except of the exogenous guidance, some endogenous stimulatory (pH, temperature, redox) materials used to engineer EVs can be effectively controlled in releasing of gene-edited components. When EVs are exposed to an acidic environment (pH 5.6–6.8), their membranes collapse and release cargo with maximum efficacy and minimum toxicity [90]. The literature on engineered EVs that deliver CRISPR/Cas systems is much less extensive. Nevertheless, other stimulus-responsive NPs regulate the delivery of the CRISPR/Cas9 system to a particular cell in a predetermined manner. For instance, zeolitic imidazolate framework-8/RNP NPs, a controlled RNP delivery method, can release up to 70% of the RNPs to the desired cell in an acidic environment or a low pH within 10 min [150, 151]. Compared with endogenous stimuli, exogenous strategies can remotely switch on/off CRISPR gene editing in real-time, enabling easy tunability, non-invasiveness, and high spatiotemporal specificity. When responding to a single stimulus, multiple-stimuli bioresponsive nanocarriers, such as pH/MF, pH/temperature, pH/redox, pH/magnetic/redox, and many other combinations, can achieve more precise and effective gene editing [150, 152]. With multiple stimuli, EV-based CRISPR/Cas9 delivery systems would provide several benefits for genome editing, such as increasing the intracellular release of EV cargo, ensuring stable encapsulation, improving editing efficiency, reducing off-target effects, and enabling well-programmed multifunctionality. However, it is limited to a few stimulating factors and remains underutilised in clinical applications. With rapid advances in implantable stimulus-responsive materials, those modification strategies can be used to deliver the EV-based CRISPR/Cas system, thereby accelerating the use of gene editing in clinical applications. Eventually, therapeutic genome editing with smart EVs could be a revolutionary delivery strategy for reaching a new milestone.

#### Chemical modification

*Covalent modification*: First, as a common method, click chemistry is applied to attach targeting moieties on the EV surface through azide–alkyne cycloaddition [143, 144] (Fig. 4a**)**. Azide–labelled cyclic peptide-EVs have been effectively provided targets treatment of ischaemic brain injury in mice [153]. Secondly, combining the metabolic engineering of parent cells with click chemistry is another strategy for producing EVs. By adding compounds, such as synthetically modified amino acids, lipids, glycans, or oligonucleotides to the cell culture medium, these compounds can be incorporated into the cell metabolism and, consequently, on the surface of the produced EVs [154]. Although click chemistry has better reaction speed, high selectivity, and compatibility, the temperature, pressure, and osmotic pressure must be carefully controlled during the modification process to avoid EV rupture and denaturation [91]. Finally, enzymatic ligation utilises protein ligases to covalently conjugate specially designed targeting moieties, such as EGFR, HER2, SIRPα-targeting peptides or their nanobodies [91], and hyaluronidase [155]. This promising EV-based platform harbours not only high-activity enzymes but also therapeutic payloads.

*Non-covalent modification*: Engineered EVs can be obtained by non-covalent modification methods, such as ligand-receptor interactions, modification of aptamers, multivalent electrostatic interactions (Fig. 4c), hydrophobic interactions/lipid insertion, and modification of anchoring peptides. Among them, the most commonly used method is lipid/hydrophobic insertion (Fig. 4b). EVs can be easily fused with functionalised liposomes embedded with targeting moieties without affecting their native function or integrity [97, 156, 157]. Next, through ligand-receptor binding, the natural receptors on the EV membrane are used to attach the targeted ligand, i.e., magnetic molecules and endosmotic peptide L17E. This enables the EVs to enhance cancer targeting under conditions of an external MF. However, this method has certain drawbacks, such as synthetic challenges and the cost of presenting functional ligands [147]. Again, as short stretches of oligonucleotide sequences or short polypeptides, aptamers are used as another type of material to functionalise EVs. Aptamer-EV conjugates can directly enter predetermined locations in the target organs, which sheds light on new practical methodologies for EV targeting [158, 159]. For specific targeted delivery of Cas9/SgRNA, the valency-controlled TDNs have been loaded on the EV surface, resulting in an increase of EV accumulation in target cells at a 1:3 ratio of aptamer/cholesterol [22]. Further, depending on multivalent electrostatic interactions, the cationised pullulan-modified EVs with positive charge reflect excellent cellular uptake for targeting the liver [160] (Fig. 4c). In some cases, the cytotoxicity of cationic nanomaterials can cause lysosomal degradation and a reduction in EV purity. Therefore, monitoring the concentration of these cationic agents is necessary to avoid their occurrence. Finally, the anchoring peptide presents a simple approach for expressing targeting moieties on the EV surface. Compared with conventional loading methods, such as transfection, electroporation, and lyophilisation, directly fusing the therapeutic moiety with the CP05 peptide can eliminate the issue of poor cargo loading; the abundance of CP05-EVs was found to be increased by 18-fold in the quadriceps using the latter approach [161] (Fig. 4d). In summary, the genetic approach may allow for a more standardised product which is desirable to address regulatory expectations. However, this strategy has several drawbacks, including changes in biological activity and challenges associated with controlling EV density. Chemical modification may offer effective control for EV surface modification both in content (preventing peptide degradation) and density of the targeting epitope, regardless of the cell source [162]. It may be performed during EV purification steps, and thus, be amenable to clinical translation. However, non-site-specific chemical modification might shield protein–protein interactions and alter EV recognition properties. It may also jeopardise the structure and function of EVs, leading to low specificity and efficiency [143, 144]. Moreover, previous studies have shown that EV subtypes exhibit different organ biodistributions and biological functions [163]. Therefore, selecting EV subtypes that display favourable targeting properties may provide new insights into the therapeutic applications of EVs.

### Artificial EVs

Artificial EVs are attractive as they closely mimic innate chemical and biological surface characteristics, such as chemical composition, membrane fluidity, and three-dimensional (3D) protein presentation. Among them, targeting specificity is a critical factor to consider in the utilization of artificial EVs in various applications. Comparatively, the natural EVs are endowed with specific surface markers and membrane proteins that confer the cell type- or tissue-specific targeting ability [123]. Given that, artificial EVs combine the advantages of natural EVs with those of NPs synthesised through different approaches [164], such as artificially synthesised EV-like NPs, EV-mimetics (EMs), HEs, and EV membrane-camouflaged NPs (Fig. 5), while their distribution is similar when biomimetic vesicles have the similar particle sizes as EVs. Based on these advantages, the functionalization of artificial EVs endows them with an enhanced tissue targeting ability, facilitates the delivery of CRISPR/Cas components payloads to the intended target site [9, 18, 49]. The common strategies for modifying artificial EVs have been well documented in previous reviews and were briefly summarised in Table 4. The large and complex units were broken down into smaller components using a top-down approach (such as extrusion through a porous membrane, sonication, and nitrogen cavitation) to prepare EV-like NPs (Fig. 5A). After the donor cells were serially extruded through the membrane filters or devices with different pore sizes, EV-like NPs were primarily produced with membrane features similar to those of donor cells [165]. Their generation achieved a 500-fold higher production yield [166] and targeting ability [167]. Conversely, EMs refer to nanovesicles synthesised using individual biomimetic molecules that resemble EV characteristics. Adopting a bottom-up strategy, small molecules can be used as building blocks to form large and complex structures through a stepwise assembly process and ultimately produce EMs (Fig. 5B). Thus, by assembling ideal components based on natural EVs, their components can be cleaned and contain controllable characteristics [168].

**Table 4:** Artificial EV preparation for targeting delivery

EV types	Principle/mechanism	Engineered strategy	EV sources	Advantages	Disadvantages	Cargo	References
Artificially synthesised EV-like NPs	Top-down strategy	Extrusion, filtration, microfluidic device, nitrogen cavitation, sonication, cell bleb, etc	RBCs, WBCs, platelets, MSCs, cancer cells, and bacteria	Simple and controllable fabrication procedure; high purity; similar size, distribution, zeta potential, and protein markers	May cause biological function loss, hard to incorporate multiple components	Therapeutic oligonucleotides, chemotherapeutic drugs	[166, 167]
EMs	Bottom-up strategy	Supramolecular chemistry	/	Easier to manufacture; safer; high yield and membrane integrity; mimicking the biological complexity of natural EVs	Low homogeneity and purity, less controllable preparation process	Drugs, therapeutic RNAs, and oligonucleotides	[168, 171]
HEs	EVs fused/hybridised with lipid membrane	Microfluidics, sonication, freeze–thaw, extrusion, hybridisation	HEK293T cells, MDA-MB-231, chondrocyte	Easy and controllable production, adjustable physical parameters, prolonged circulation time	May lose biological functions of EVs, difficult fabrication and purification, low homogeneity and yield	CRISPR/Cas9 plasmid, therapeutic RNAs, chemotherapeutic drugs	[9, 18, 49]
EV membrane-camouflaged NPs	EV membrane encapsulating inorganic/organic NPs	/	MSCs, neutrophil	Maintain the complex structure of EVs, specific targeting, immune escape, high therapy efficacy	Low scalability, difficult fabrication, time-consuming	Proteins, therapeutic RNAs, bioactive lipid mediators, imaging agents	[105, 170, 172]

HEs have been used to optimise loading, immune evasiveness, and ability to cross biological barriers (Fig. 5C). As stated earlier, liposome-fused HEs have successfully delivered the CRISPR/Cas9 system to the target cells to achieve more effective gene editing efficiency [9, 18, 49]. HEs can also be prepared by fusing two different original parent cell membranes, which can synergistically perform the complex activities of both cells. HEs exhibit potential for large-scale production [169]. EV membrane-camouflaged NPs are typically prepared using an active EV membrane to encapsulate inorganic or organic NPs (Fig. 5D). The coated NPs protect the loaded cargo from immune clearance and promote the targeted release of intracellular cargo [105]. However, NPs may induce an immune response in vivo after washing out the membrane camouflage. Thus, neutrophil membrane-derived EVs can directly load therapeutic elements without NPs [170]. These strategies provide attractive prospects for expanding the applications of EVs beyond their original functions. Although artificial EVs have many advantages and gradually applied to deliver the CRISPR/Cas components, several caveats should be considered when selecting an appropriate method, including cell source, cargo type, and immune response.

## Tracking

Tracking techniques can provide valuable insights into the distribution, transport, and targeting properties of EVs used for delivering gene-editing components in vivo. By labelling the molecules or components within EVs, their locations and fates can be tracked to assess their delivery efficiency and targeting precision in vivo. To date, hundreds of heterogeneous markers, such as fluorescent, bioluminescent, and radioactive tracers, have been developed for EV tracking [173]. Each marker offers different sensitivities, times, and spatial resolution. EVs are labelled with these markers primarily through chemical modifications, such as click chemistry [174] (Fig. 4a), metabolic glycoengineering [175], hydrophobic insertion [176], direct fusion [30] (Fig. 4b and e), and genetic engineering [177] (Fig. 4A). EV modification with tracers, such as fluorescent dye PKH26/67, DiR/DiO, Cy5.5 [7, 22, 30, 38], or radioactive agent 111In-oxine [92, 125, 174, 178], provides EV-based CRISPR/Cas components with the desired functionality without changing EVs size, internalisation pattern, and properties (Fig. 4). Through imaging of organs and analysis of tissue lysates in vitro, ^111^In-oxine and DiR were found to be the most sensitive tracers for EV imaging in vivo, providing the most accurate quantification of EV biodistribution. Radioactive traces are the most accurate EV tracking approach for a complete quantitative biodistribution study and pharmacokinetic profiling [173, 179]. All these techniques possess a distinctive advantage and can be used as a criterion for selecting labelling methods depending on the purpose of EV tracking/imaging.

Through genetic manipulation, the fusion of luciferase and CD63 alters the distribution of EVs, resulting in high their accumulation in lungs [173]. This implies that EVs engineered for tracking purposes might compromise their physiological biodistribution. True single-molecule fluorescence optimised using the CRISPR/Cas9 method has been studied as an improved quantitative analysis of individual EV localisation, distribution, and uptake to avoid the potential artefacts caused by marker overexpression. The results showed that a single CRISPR/Cas9 GFP-CD63-labelled EV further improved the quantitative analysis of EV biological distribution (83%) compared to overexpression of GFP-CD63 (36%) [177]. Furthermore, used a different tactic to visualise the function of CRISPR/Cas9 components, the results showed that EVs-based-CRISPR tracking system can more efficiently abrogate the target gene in recipient cells [43]. To improve the efficiency and sensitivity of this tracking system, the sgRNA could be optimized to trigger the expression of reporter protein more efficiently [64]. In contrast to genetic engineering, pH-reversible boron dipyrromethene fluorescent probes have been used to track EV imaging without severe cytotoxicity. When acidic EVs or their precursors are encountered for ‘always-on’ dyes, pH transition empowers their imaging with minimal false positive signals [180].

Although fluorescence or luminescence imaging techniques are readily available and the instruments are easy to operate, they do not offer high sensitivity and absolute quantification. Live imaging single-photon emission computed tomography (SPECT), positron-emission tomography, and anatomical computed tomography/magnetic resonance imaging tracking overcome these limitations and provide radiolabelled EVs. They offer higher sensitivity, absolute quantification, and the advantages of non-invasive techniques, which help understand the functions of EVs in the physiology and pathophysiology of diseases. As imaging modalities can provide information about the therapeutic dose of EVs and potential side effects, the pharmacokinetics and biological behaviour of EVs could be favourable in fostering improved diagnosis and treatment of many diseases [179]. Nevertheless, SPECT imaging technology is limited owing to the shortcomings of relatively long acquisition times, low spatial resolution, and changes in the propriety of EVs in practical applications. Given that near-infrared fluorescence (NIRF) imaging offers the advantages of real-time and high-resolution, multimodality SPECT-NIRF imaging provides more accurate spatial positioning and 3D information for detecting small lesions [181]. Thus, the modified EVs could be used as carriers for multimodal imaging, which could open up a new treatment avenue in precision medicine [176, 182].

## Conclusions and future perspectives

In the past decade, the use of the CRISPR/Cas system, a revolutionised and powerful genome editing tool, has been growing expeditiously worldwide across various fields. When delivering the CRISPR/Cas components to the target sites, an appropriate carrier is a prerequisite for ensuring their safety, efficacy, and specificity. Using various design or modification strategies, engineered EVs could be an effective tool for gene delivery. However, delivery of CRISPR/Cas systems with EVs remains in its infancy, and extensive progress is needed before EV-based vectors can compete with their more time-honoured counterparts. In particular, certain issues must be addressed, (I) Currently, there is a lack of the guidelines for standardised operation for EV/engineered EV large-scale manufacturing, isolation, characterisation, storage, dosage, and functionality assessment. Some strategies, such as building donor cell banks [183–185], enhancing EV yield by different stimulation [186], producing artificial EVs (Fig. 5), using hollow-fibre bioreactors and 3D-printed scaffolds [187, 188], incorporating separation technologies (microfluidics, centrifugal [189], acoustical forces [190], and filtration combined with anion exchange [191]), could be suitable for preparing EVs for clinical applications following large-scale good manufacturing practice grade. (II) Regarding cargo delivery, multiple aspects, including the interaction of EV-loaded exogenous cargo and endogenous cargo, unclear pharmacokinetics and biodistribution pattern profiles of delivery systems in vivo, imperfect EV biomarkers and their reference range, and the balancing mechanism of EV processing and degradation must be explored. (III) The choice of an appropriate administration route, such as systemic [96] or local injection [21], is crucial to the facilitate preclinical and clinical transformation of EVs. Other potential administration routes, including inhalation, oral, local infiltration, or optimised EV-based spatiotemporal control of delivery routes, warrant further exploration in future studies. (IV) The distribution and degradation of engineered EVs in vivo must be studied. (V) Considering that the pathogenesis of each disease and the delivery barriers of each target cell differ, developing versatile delivery systems capable of delivering all three forms of CRISPR/Cas components into multiple target cells is not recommended.

A growing body of literature has demonstrated that numerous diseases are associated with structural or functional changes in various subcellular organelles, such as mitochondria [192], endoplasmic reticulum [193], Golgi apparatus, and lysosomes [194]. Many optimized gene editing tools have been established to edit the organelles of mitochondria [195], lysosomes [196], and chloroplast [197], such as the Cas 13a, base editors and prime editors [198]. With the exploitation of EVs secreted by organelles [199], or with the assistance of biomimetic technology [200], targeting the delivery of CRISPR/Cas components to organelles with EVs would suggest a significant research direction for EV-based gene fixed-point editing applications. Presently, our team is constructing a novel expression system by targeting the delivery of the CRISPR/Cas9 system with EVs/NPs into the nucleus/chloroplast of *Dunaliella salina* [201, 202]. Establishing nucleus-chloroplast dual expression systems in *Dunaliella salina* has important practical significance for the large-scale production of foreign proteins. Despite EV-mediated gene editing still facing multiple technical hurdles, as discussed previously, following the resolution of these fundamental issues, EV-based CRISPR/Cas delivery systems show great promise for safe and efficient gene editing, which would soon realise their full potential in numerous fields.

## Data Availability

All data of this article are included within the article. It can also be requested from the corresponding author or first author.

## Abbreviations

EVs: Extracellular vesicles
MVEs: Multivesicular endosomes
MVs: Microvesicles
CRISPR: Clustered regulatory interspaced short palindromic repeats
Cas9: CRISPR-associated protein 9
sgRNA: Synthetic guide RNA
RNP: Ribonucleoprotein
RNPs: Ribonucleoprotein complexes
pDNA: Plasmid DNA
NPs: Nanoparticles
DCs: Dendritic cells
RBCs: Red blood cells
WBCs: White blood cells
VLPs: Virus-like particles
eVLPs: Engineered VLPs
ABP: Aptamer-binding protein
HEs: Hybrid EVs
TDNs: Tetrahedral DNA nanostructures
TNF-α: Tumour necrosis factor α
HuR: Human antigen R
SEAL: Sonication and extrusion-assisted active loading
EPNs: Enveloped protein nanocages
IVT: In vitro transcribed
RES: Reticuloendothelial system
PEG: Polyethylene glycol
DSPE: Distearoyl phosphatidyl ethanolamine
DMPE: Dimyristol phosphatidyl ethanolamine
ARRDC1: Protein-1 trap domain
SIRPα: Signal regulatory protein α
PS: Phosphatidylserine
C1C2: Lactadherin
RGD: Arg-Gly-Asp
EGFR: Epidermal growth factor receptor
MSCs: Mesenchymal stem/stromal cells
VSV-G: Vesicular stomatitis virus glycoprotein
Lamp2B: Lysosome-associated membrane glycoprotein 2
PLGA: Polylactic-co-glycolic acid
GPI: Glycosyl-phosphatidyl-inositol
PDGFR: Platelet-derived growth factor receptors
GFP: Green fluorescent protein
MHC: Major histocompatibility complex
MF: Magnetic field
SPECT: Single-photon emission computed tomography
NIRF: Near-infrared fluorescence
EMs: EV-mimetics
Ig: Immunoglobulin
PTX: Paclitaxel
3D: Three-dimensional
pH: Pondus hydrogenii
CIBN: CRY-interacting basic-helix-loop-helix 1
CRY2: Cryptochrome 2
MC DNA: Comprising engineered minicircle MC DNA
ACE2: Angiotensin-converting enzyme 2
tLyp-1 peptide: Amino-acid sequence CGNKRTR
